# Selective Antimicrobial and Cytotoxic Effects of Hop Compounds to Combat Poultry Antimicrobial Resistance

**DOI:** 10.1002/mbo3.70080

**Published:** 2025-10-21

**Authors:** Luisa Kober, Kathrin Castiglione

**Affiliations:** ^1^ Institute of Bioprocess Engineering Friedrich‐Alexander‐Universität Erlangen‐Nürnberg Erlangen Germany

**Keywords:** antibiotic resistance, antimicrobials, *Humulus lupulus*, phytogenics, UMNSAH/DF‐1

## Abstract

The rising global population has led to an increased demand for poultry meat, necessitating the development of sustainable and safe alternatives to antibiotic growth promoters, which have been banned or restricted due to concerns about antibiotic resistance. Phytogenic feed additives (PFAs), particularly hop‐derived compounds, have shown promise as natural antimicrobial agents. This study investigates the antibacterial and cytotoxic properties of five major hop‐derived compounds —isoxanthohumol, xanthohumol, humulone, lupulone, and isohumulone —to assess their potential as PFAs in poultry farming. Using broth dilution and metabolic activity assays, minimum inhibitory concentrations (MIC_50_) and cytotoxic concentrations (IC_50_) were determined against *Micrococcus luteus*, *Bacillus subtilis*, and a chicken fibroblast cell line (UMNSAH/DF‐1). Among the compounds tested, xanthohumol demonstrated the most favorable selective index against *M. luteus* (5.5 ± 0.36), while for *B. subtilis*, lupulone had an even higher index (15 ± 0.77). These findings highlight the novel potential of specific hop‐derived compounds as selective and safe PFAs, contributing valuable insights into natural alternatives to conventional antibiotic growth promoters.

## Graphical abstract

Phytogenic feed additives (PFAs) like hop (*Humulus lupulus*) offer a sustainable alternative to antibiotics in the expanding global poultry market. This study tested five hop‐derived compounds for antibacterial activity against *Micrococcus luteus* and *Bacillus subtilis*, and for their cytotoxicity in chicken cells. Xanthohumol showed the highest selectivity index (5.5 ± 0.36) for *M. luteus*, while lupulone had an even higher index (15 ± 0.77) for *B. subtilis*. Hop compounds are therefore promising candidates for sustainable PFAs in poultry farming.

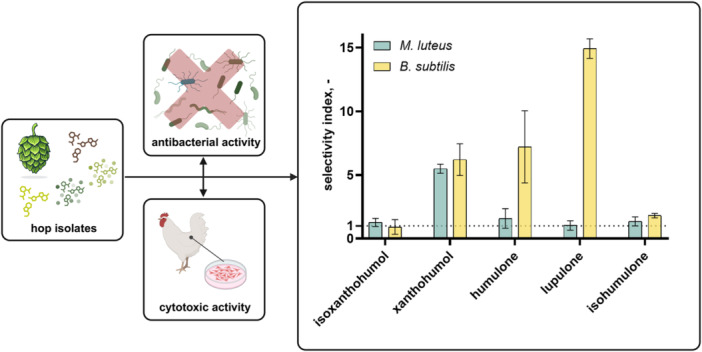

## 1 Introduction

The global production and consumption of meat is on the rise, with poultry meat representing the largest share at 40% of the total. Poultry meat offers numerous advantages, including its acceptance across diverse cultural groups and its favorable nutritional profile, characterized by high protein and low fat content (Kober et al. 2024). However, as consumption increases, it is imperative that the production of poultry adheres to a sustainable concept. The implementation of effective health management and disease prevention strategies is crucial for the enhancement of poultry welfare and sustainability (Bist et al. 2024). One of the most significant challenges in the context of poultry health is the emergence of antimicrobial resistance (AMR). The excessive use of antimicrobials in animal production is a contributing factor. It is postulated that the indiscriminate use of antimicrobials in animal farming will accelerate the development of AMR in pathogens, as well as in commensal organisms (Nhung et al. 2017). It is therefore unsurprising that the prophylactic use of antibiotics was (at least partially) restricted or even banned in all EU countries, the USA, Canada, and the UK (Salim et al. 2018).

**Figure 1:**
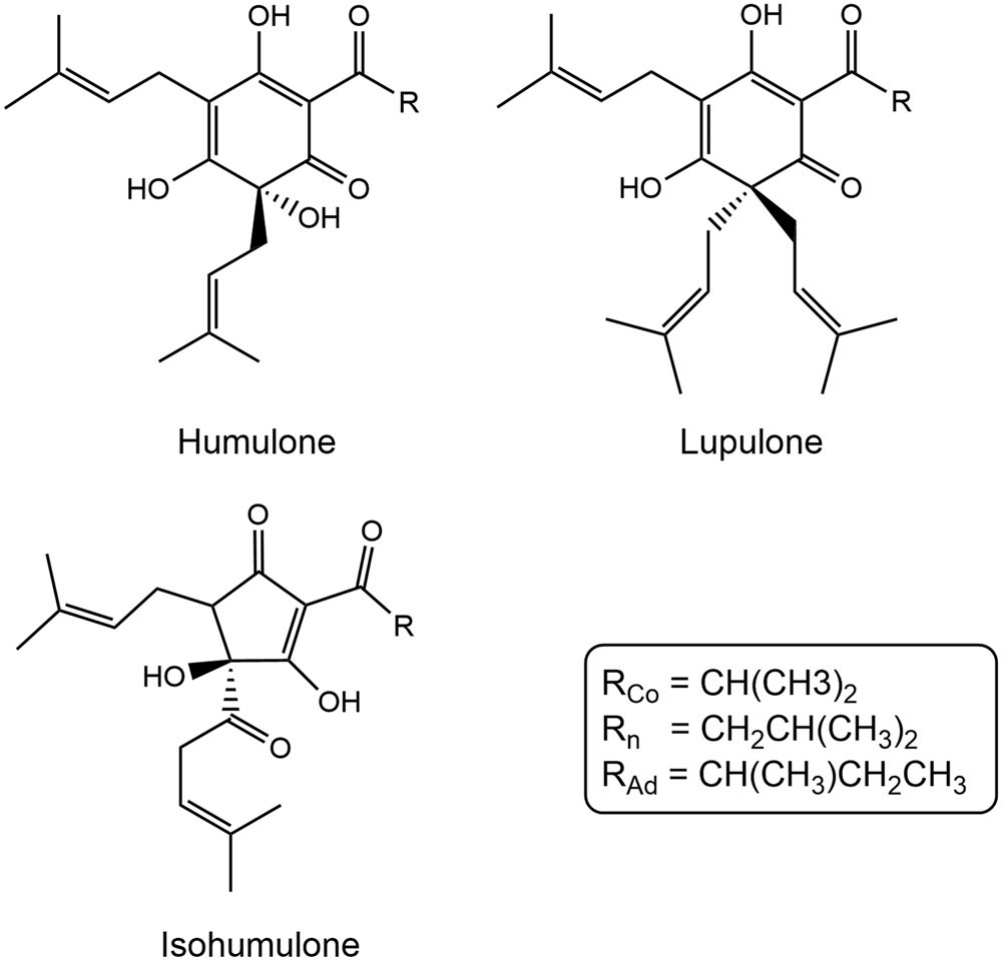
Hop bitter acids humulone and lupulone, as well as isomerized isohumulone. Co‐/n‐ and Ad‐derivatives form the largest part out of all derivatives (Karabín et al. 2016).

To maintain the health status of poultry without the prophylactic use of common antibiotics, alternative substances must be employed to combat AMR. One potential solution is the administration of phytogenic feed additives (PFAs) instead of classic antibiotics. PFAs are defined as natural, plant‐derived substances that have been demonstrated to enhance animal health and increase production performance (Wang et al. 2024). A possible candidate for utilization in poultry farming is the common hop, *Humulus lupulus* (*H. lupulus*), which has predominantly been applied in the context of beer brewing. In this context, the bitter acids (resins) are of significant importance, as they are responsible for the distinctive flavor and shelf life of the beer (Kober et al. 2024). The bitter acids (Figure 1), which can be divided into alpha‐ (humulone) and beta‐ (lupulone) bitter acids, display hydrophobic properties. Consequently, it is primarily the less hydrophobic isohumulones, which are isomerized by temperature, that can pass into the wort (Karabín et al. 2016). The extension of shelf life is a result of the antibacterial effects of humulones, lupulones, and isohumulones. These compounds have demonstrated efficacy in combating Gram‐positive bacterial pathogens, such as *Bacillus*, *Micrococcus*, *Staphylococcus*, *Mycobacterium*, and *Streptomyces* (Bocquet et al. 2018). The inhibitory effects of hop extracts even extend to methicillin‐resistant *Staphylococcus aureus*, one of the most common healthcare‐associated infections in the USA (Wendakoon et al. 2019). The underlying principle is that hop bitter acids permeate bacterial cell membranes, thereby causing leakage. This results in the effective inhibition of membrane transport, enzyme functions, and nutrient intake, particularly in Gram‐positive bacteria (Korpelainen and Pietiläinen 2021), which can be attributed to the distinctive structure of their thick cell wall (Beveridge 2001).

**Figure 2:**
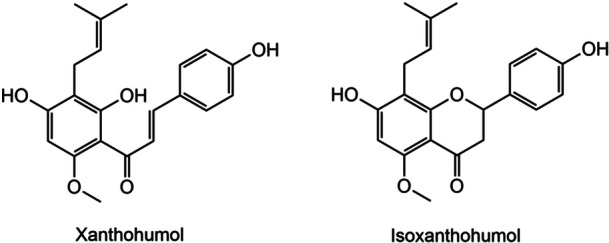
Polyphenolic compounds xanthohumol and its isomerized form isoxanthohumol (Karabín et al. 2016).

It is not only the bitter acids that have antimicrobial effects, but also the polyphenols. Polyphenols, such as xanthohumol (Figure 2), inhibit the replication of several microorganisms by accumulating inside cells or penetrating the phospholipid cell membranes, mostly of Gram‐positive bacteria. Due to their relatively hydrophobic character, prenylflavonoids, of which xanthohumol is an example, have attracted increasing attention in scientific research over recent years (Karabín et al. 2016).

While the antibacterial properties of hops have been the subject of some studies (e.g., Fahle et al.), there is a lack of literature on cytotoxic effects. Furthermore, the extant literature contains no studies that compare all components simultaneously to analyze the correlation effects of the named isolates. However, for use as a feed additive in poultry farming, it is important that these effects are better understood. To assess the effects of the different hop compounds, selectivity indices can be calculated that relate the antibacterial effect to the cytotoxic effect. Therefore, we investigated the different inhibitory effects of hop compounds humulone, lupulone, isohumulone, xanthohumol, and isoxanthohumol on two bacterial strains and a chicken cell line to find out which of these compounds has the best selectivity indices. On the basis of these results, conclusions can be drawn as to which hop compounds should be enriched in hop extracts to produce a sustainable product for poultry farming based on the raw material hop.

## 2 Materials and Methods

### 2.1 Bacterial Strain, Media, and Culture Conditions

*Micrococcus luteus* (DSM 1605) was obtained from DSMZ (German Collection of Microorganisms and Cell Cultures GmbH). It was cultured on nutrient agar or in nutrient broth (NB, DSMZ #1) containing 5 g L^−1^ peptone from casein (Carl Roth, Karlsruhe, Germany) and 3 g L^−1^ meat extract (Carl Roth, Karlsruhe, Germany) under aerobic conditions at 37°C.

*Bacillus subtilis* (DSM 23778) was obtained from DSMZ (German Collection of Microorganisms and Cell Cultures GmbH). It was cultured on tryptic soy broth (TSB, DSMZ #545) agar plates or in TSB containing 17 g L^−1^ peptone from casein, 3 g L^−1^ peptone from soy, 2.5 g L^−1^ d‐(+)‐glucose, 5 g L^−1^ NaCl, and 2.5 g L^−1^ K_2_HPO_4_ (Carl Roth, Karlsruhe, Germany) under aerobic conditions at 28°C.

### 2.2 Chicken Cell Line, Media, and Culture Conditions

UMNSAH/DF‐1 cell line was purchased from Cytion (Eppelheim, Germany). The cells were maintained in Dulbecco's Modified Eagle Medium (DMEM, Sigma‐Aldrich, St. Louis, USA) containing 10% heat‐inactivated fetal bovine serum (FBS, Sigma‐Aldrich, St. Louis, USA) and cultured at 37°C and 5% CO_2_ in a humidified atmosphere. Cell lines were serially passaged after being detached with Accutase solution (Sigma‐Aldrich, St. Louis, USA).

### 2.3 Chromatographic Analysis of Hop Compounds

High‐performance liquid chromatography (HPLC) measurements were performed using an NUCLEODUR C18 Gravity‐SB (3 µm, 250 × 2 mm) column equipped with a precolumn (Machery Nagel, Düren, Germany) in a Shimadzu HPLC system (Kyoto, Japan). The flow rate was set to 0.6 mL min^−1^ at 40°C using 80% methanol/20% phosphoric acid (0.5% [v/v] in H_2_O) as mobile phase. Media samples were 10‐fold diluted in the mobile phase, incubated for 10 min at 40°C and centrifuged at 10,000*g* for 10 min. 10 μL of filtered (polytetrafluoroethylene, 0.2 µm) samples was injected for analysis. Identification and quantification of isoxanthohumol, xanthohumol, co‐/n‐/adhumulone, co‐/n‐/adlupulone, and co‐/n‐/adisohumulone by a UV‐Detector (314 nm for 0–24 min, and 270 nm for 24.01–45 min) were performed by corresponding standard curves. The retention times are shown in Table 1.

**Table 1:** Retention times for hop compounds under the described conditions.

Compound	Retention time, min
Isoxanthohumol	3.0
Xanthohumol	4.8
Cohumulone	12.0
n‐/Adhumulone	15.0
Colupulone	17.6
n‐/Adlupulone	22.6
Isocohumulone	25.1
iso‐n‐Humulone	28.0
Isoadhumulone	29.6

### 2.4 Hop Compound Preparation in Aqueous Media

The preparation of hop compound solutions was carried out analogously for all three media NB, TSB, and DMEM for use in bacterial and avian cell culture. Hop compounds were provided by Hopsteiner (Mainburg, Germany) in the following purities: isoxanthohumol (81.0%), xanthohumol (90.0%), humulone (88.5%), lupulone (74.3%), and isohumulone (91.0%). The hop compounds were dissolved in ethanol to 10 mg mL^−1^. From this stock solution, 1:10 dilutions were prepared in the respective medium, resulting in precipitation due to their hydrophobic character. After centrifugation (10,000*g*, 10 min), the clear supernatant was transferred to a new tube and used for further experiments. Due to the low solubility of the compounds, the actual dissolved concentration was in most cases lower than the weighed concentration. This is why the actual concentration of the compounds in the aqueous medium was analyzed using HPLC and used for subsequent calculations.

### 2.5 Antibacterial Activity

Antibacterial activity of hop compounds against Gram‐positive *M. luteus* and *B. subtilis* was analyzed using Broth Dilution Assay. Twofold dilutions of hop compounds were prepared in NB or TSB medium, ranging from theoretical concentrations of 500 to 0.5 µg mL^−1^ and transferred to 48‐well microtiter plates (Sarstedt, Nuembrecht, Germany). Gentamicin sulfate was used as a positive control. The optical density (OD) of a preculture prepared on the previous day was measured at 600 nm in a spectrophotometer (Implen, Muenchen, Germany). The inoculum was then diluted with NB or TSB medium to the desired OD_600 nm_ of 0.1 and transferred to all wells (150 µL) except the sterile control, which consists only of medium. The ODs of the cultures were measured every hour at 750 nm using a plate reader (Tecan, Maennedorf, Switzerland) for 24 h. In the meantime, the plates were incubated at 37°C or 28°C without shaking. The chosen wavelength of 750 nm resulted from previous experiments with hop extracts to avoid an overlap of the absorption spectrum with chlorophyll (Guidi et al. 2017). The growth rates were obtained from the exponential range of the generated growth curves. All values were normalized to the growth control (without hop compounds). MIC_50_ (minimum concentration of a substance that inhibits the growth of 50% of the bacteria tested) values were calculated by nonlinear regression using GraphPad Prism model “log(inhibitor) versus response,” where the basal response was subtracted and the bottom was set to a constant value (0). Means and standard deviations (SDs) were calculated from two biological duplicates, each consisting of three technical replicates.

### 2.6 Cytotoxic Activity

The hop compounds were investigated for their cytotoxic effect on the chicken cell line UMNSAH/DF‐1, using the 3‐(4,5‐dimehtylthiazol‐2‐yl)‐2,5‐diphenyltetrazolium bromide (MTT) based cell viability assay. Cells were seeded in 96‐well microtiter plates (Sarstedt, Nümbrecht, Germany) with a cell density of 0.05 × 10^6^ cells per mL and 100 µL per well and incubated for 24 h (37°C, 5% CO_2_). After that the medium was discarded and a dilution series of hop compounds in DMEM was added to the wells (100 µL), ranging from theoretical concentrations of 200 µg mL^−1^ to 20 ng mL^−1^. For vitality control, 20% dimethyl sulfoxide (DMSO) (TH Geyer, Renningen, Germany) was added, and for growth control, culture medium was added. The cytotoxic measurements were also performed with gentamicin sulfate as a control. Treated cells were further incubated for 48 h (37°C, 5% CO_2_). Then, 12.5 µL per well of MTT (Sigma‐Aldrich, St. Louis, USA) solution (0.5% [w/v]) in phosphate‐buffered saline was added, followed by another 3 h of incubation (37°C, 5% CO_2_). The plates were centrifuged (300*g*, 5 min), the supernatant was discarded, and 20 µL of Igepal (0.4% (v/v) in H_2_O (Sigma‐Aldrich, St. Louis, USA) was added. After incubation on the plate shaker for 10 min, 100 µL per well of DMSO was added to dissolve the formazan. The plates were incubated again on the plate shaker for 30 min. Absorbance at 570 nm were measured using a plate reader (PerkinElmer, Waltham, USA). The resulting absorbance was directly proportional to the amount of viable cells. All values were normalized to the growth control. IC_50_ (concentration of a substance that inhibits the metabolic activity of 50% of the cell line tested) values were calculated analogously to MIC_50_ values. Means and SDs were calculated from two biological duplicates, each consisting of three technical replicates.

## 3 Results

### 3.1 Solubility of Hop Compounds in an Aqueous Media

**Figure 3:**
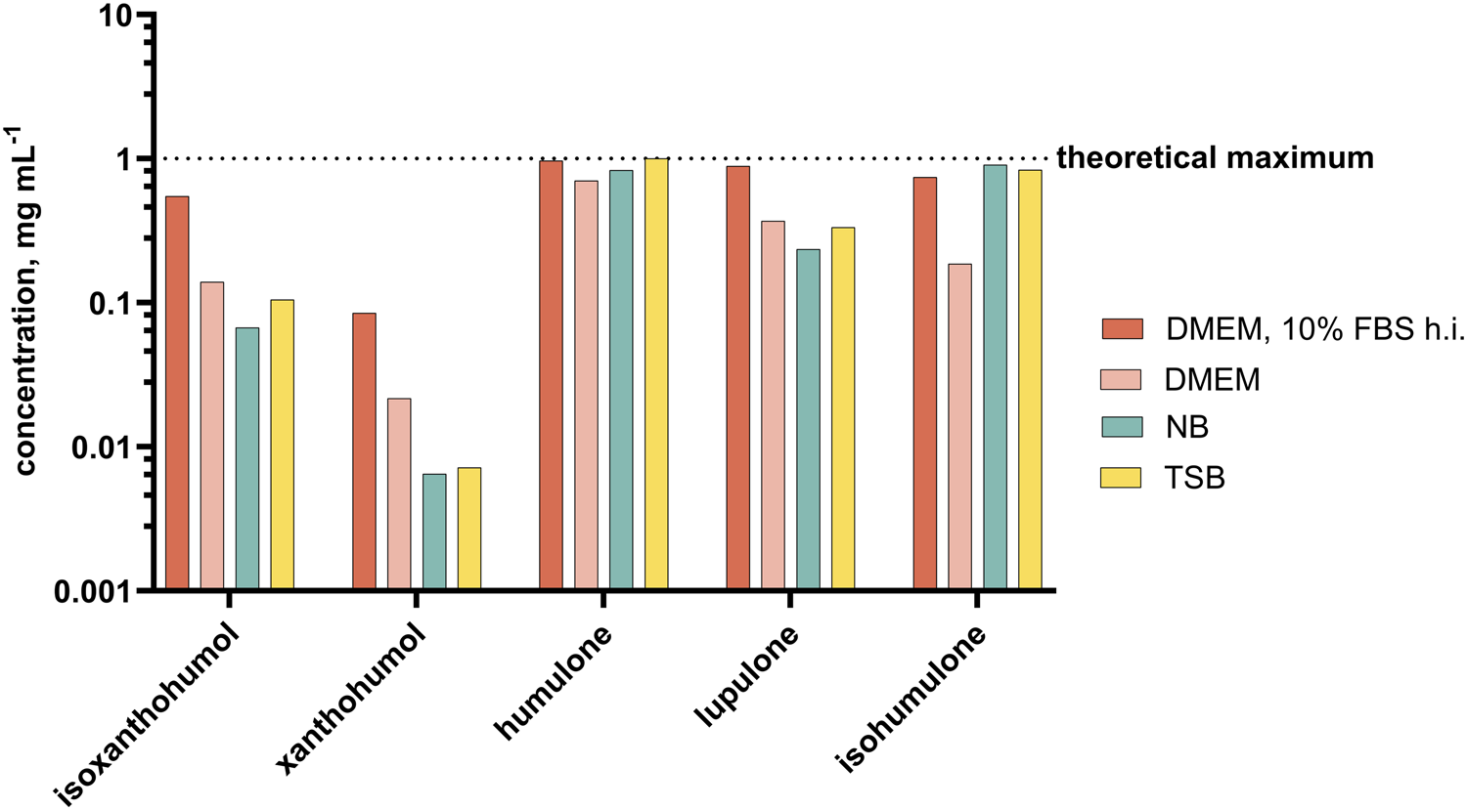
Solubility of hop compounds in three aqueous media, DMEM with and without 10% FBS h.i., NB and TSB, with a final ethanol concentration of 10% (v/v). Concentrations were determined from supernatants after centrifugation via HPLC analysis. DMEM, Dulbecco's Modified Eagle Medium; FBS, fetal bovine serum; HPLC, high‐performance liquid chromatography; NB, nutrient broth; TSB, tryptic soy broth.

Due to the hydrophobic character of hop pure compounds, a complete dissolution in an aqueous medium cannot be assumed. This was demonstrated by the precipitation of the pure substances when the ethanolic stock solution was diluted in an aqueous medium. Figure 3 shows the individual concentrations of five hop compounds in the three used media, DMEM with 10% FBS h.i., NB, and TSB, after dissolution of 10 mg mL^−1^ ethanolic stock solution in the desired medium to a theoretical concentration of 1 mg mL^−1^. To assess the influence of FBS on solubility, DMEM without serum was also tested. For humulone, lupulone, and isohumulone, the three derivatives (co‐/n‐/ad‐) are considered together in this figure and also in the following.

The dissolution in DMEM with 10% FBS h.i. proved to be more efficacious than dissolution in NB or TSB media. The addition of serum resulted in enhanced solubility; however, serum‐free media exhibited higher solubility than bacterial NB or TSB media for most compounds. The solubility in aqueous medium was highest for humulone and isohumulone, whereas it was minimal for xanthohumol and isoxanthohumol. The actual concentrations of the compound solutions determined this way were then used to calculate MIC_50_ for antibacterial and IC_50_ for cytotoxic activity.

### 3.2 Antibacterial Activity of Hop Compounds Against *M. luteus* and *B. subtilis*

**Figure 4:**
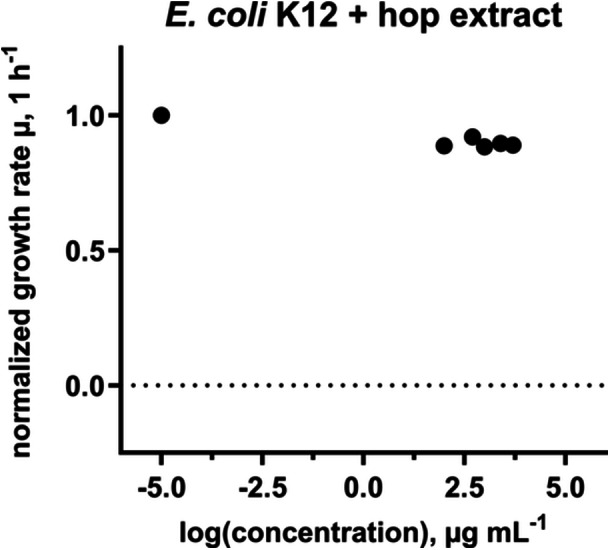
Influence of hop extracts on the growth of Gram‐negative *Escherichia coli* K12. The leftmost data point represents the growth control without hop extract. Hop extract had no effect on growth inhibition in *E. coli* K12.

**Figure 5:**
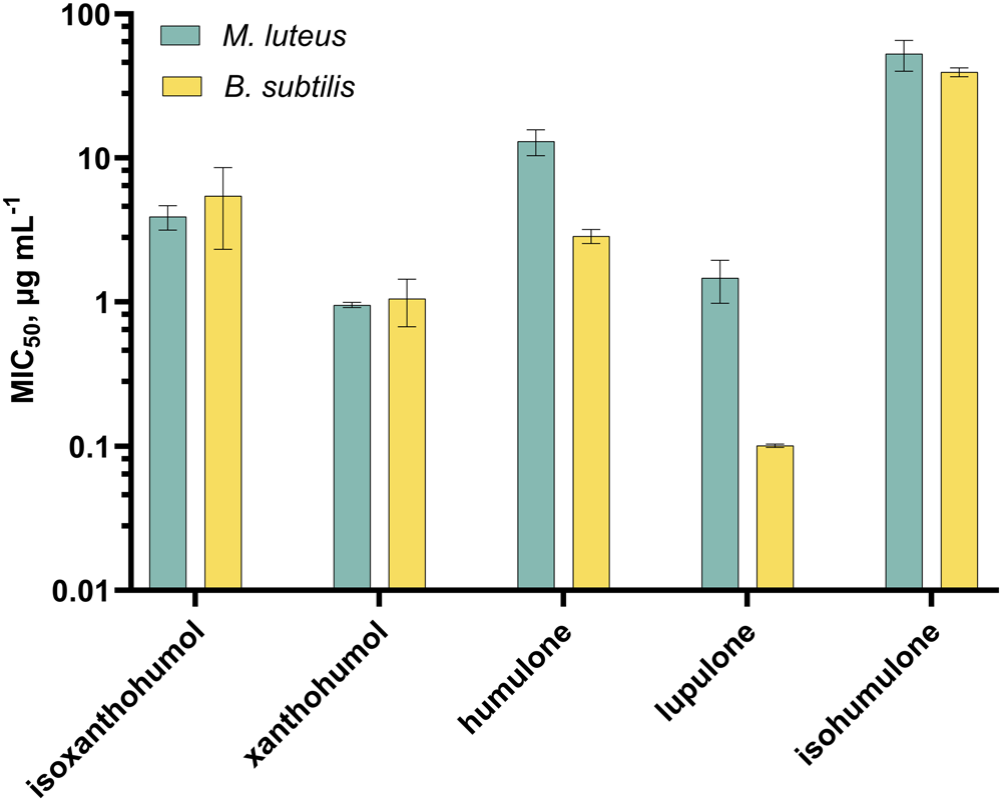
Antibacterial activity of five hop compounds against Gram‐positive *Micrococcus luteus* and *Bacillus subtilis* determined via broth dilution assay. MIC_50_ are biological duplicates. MIC_50_, minimum inhibitory concentrations.

**Figure 6:**
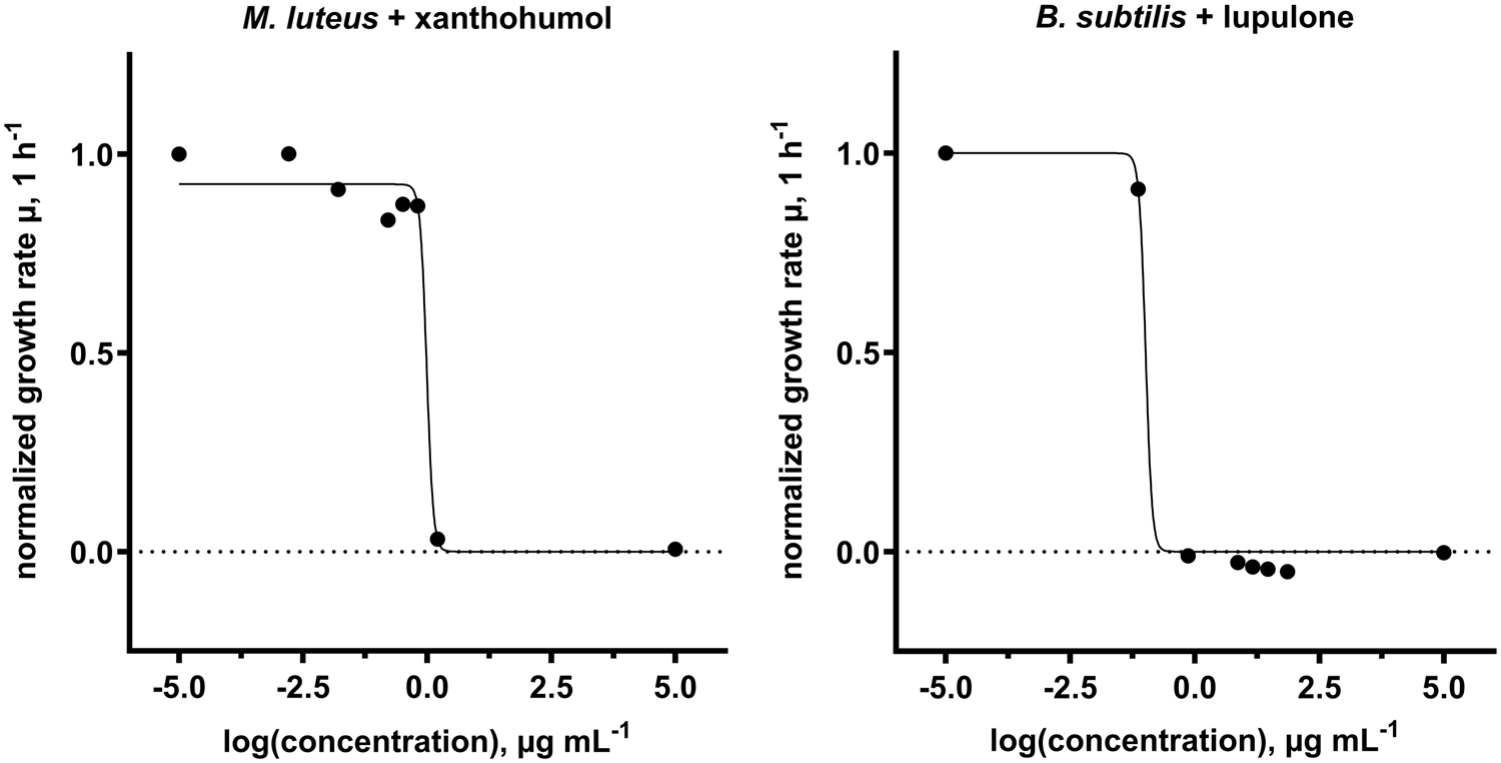
Exemplary dose response curves for *Micrococcus luteus* (*R* ^2^ = 0.9863) and *Bacillus subtilis* (*R* ^2^ = 0.9953) with their respective strongest antibacterial acting hop isolate. The growth control is displayed as “‐5” representing a concentration near 0, whilst the sterile control is displayed as “5” representing a highly antibacterial concentration.

Expectedly, previous tested hop extracts were not effective against the Gram‐negative bacterial strain *Escherichia coli* K12 (Figure 4). Therefore, the investigation of the antibacterial effect of pure hop compounds was focused on the Gram‐positive bacterium *M. luteus* and *B. subtilis*. Results are shown in Figures 5 and 6. MIC_50_ describes the concentration at which half of the bacterial growth was inhibited by a given compound. All compounds showed antibacterial activity against the bacteria tested, but the five isolates showed different responses to the two bacterial strains. For *M. luteus*, xanthohumol demonstrated the strongest inhibitory effect (MIC_50_ = 0.95 ± 0.040 µg mL^−1^), followed relatively closely by lupulone (MIC_50_ = 1.5 ± 0.48 µg mL^−1^), while for *B. subtilis* lupulone (MIC_50_ = 0.10 ± 0.0029 µg mL^−1^) had by far the strongest antibacterial activity. Isohumulone demonstrated the least potent overall inhibitory effect for both bacterial strains with MIC_50_ values over 40 µg mL^−1^. In comparison, gentamicin sulfate exhibited MIC_50_ similar values of 0.26 ± 0.08 µg mL^−1^ (*M. luteus*) and 0.73 ± 0.20 µg mL^−1^ (*B. subtilis*).

### 3.3 Cytotoxic Activity of Hop Compounds on UMNSAH/DF‐1

**Figure 7:**
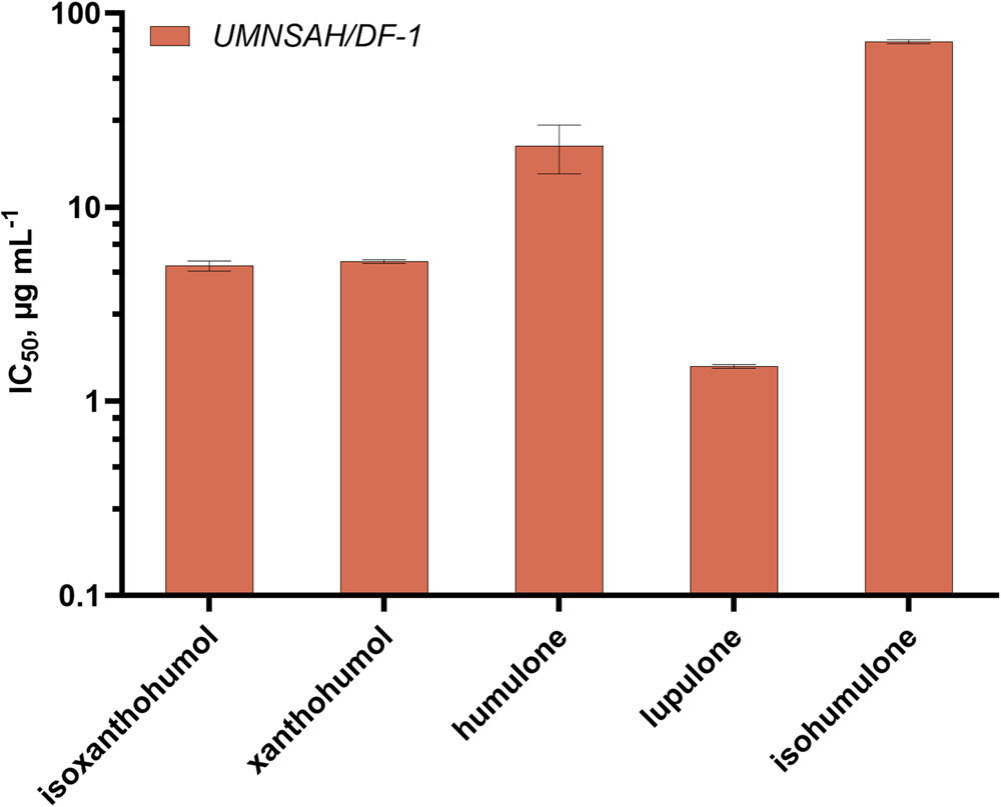
Cytotoxic activity of five hop compounds against the chicken cell line UMNSAH/DF‐1 determined via MTT assay. IC_50_ values are biological duplicates. IC_50_, inhibitory concentration; MTT, 3‐(4,5‐dimehtylthiazol‐2‐yl)‐2,5‐diphenyltetrazolium bromide.

**Figure 8:**
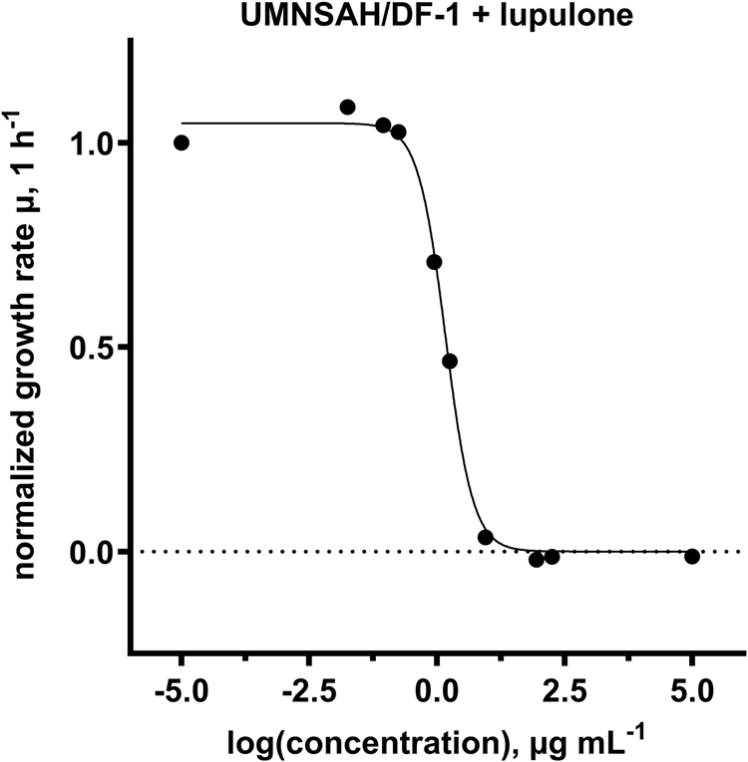
Exemplary dose response curves for UMNSAH/DF‐1 (*R* ^2^ = 0.9804) and hop isolate lupulone. The growth control is displayed as “‐5” representing a concentration near 0, whilst the sterile control is displayed as “5” representing a highly cytotoxic concentration.

To be able to use hops as an antibiotic alternative in poultry farming, the cytotoxic effect must be taken into account in addition to the antibacterial effect. For this reason, the chicken cell line UMNSAH/DF‐1 was used for a cytotoxicity study. Results are shown in Figures 7 and 8. IC_50_ describes the concentration at which half of metabolic activity was inhibited by a given compound. All compounds showed cytotoxic effects against the tested cell line. Lupulone (1.5 ± 0.034 µg mL^−1^) showed the strongest effect, whilst isohumulone (71 ± 1.8 µg mL^−1^) showed only moderate to weak cytotoxic properties compared with the other compounds. The cytotoxic effect of gentamicin sulfate was much weaker, with an IC_50_ of 1.44 ± 0.39 mg mL^−1^.

### 3.4 Selective Effect of Hop Compounds

**Figure 9:**
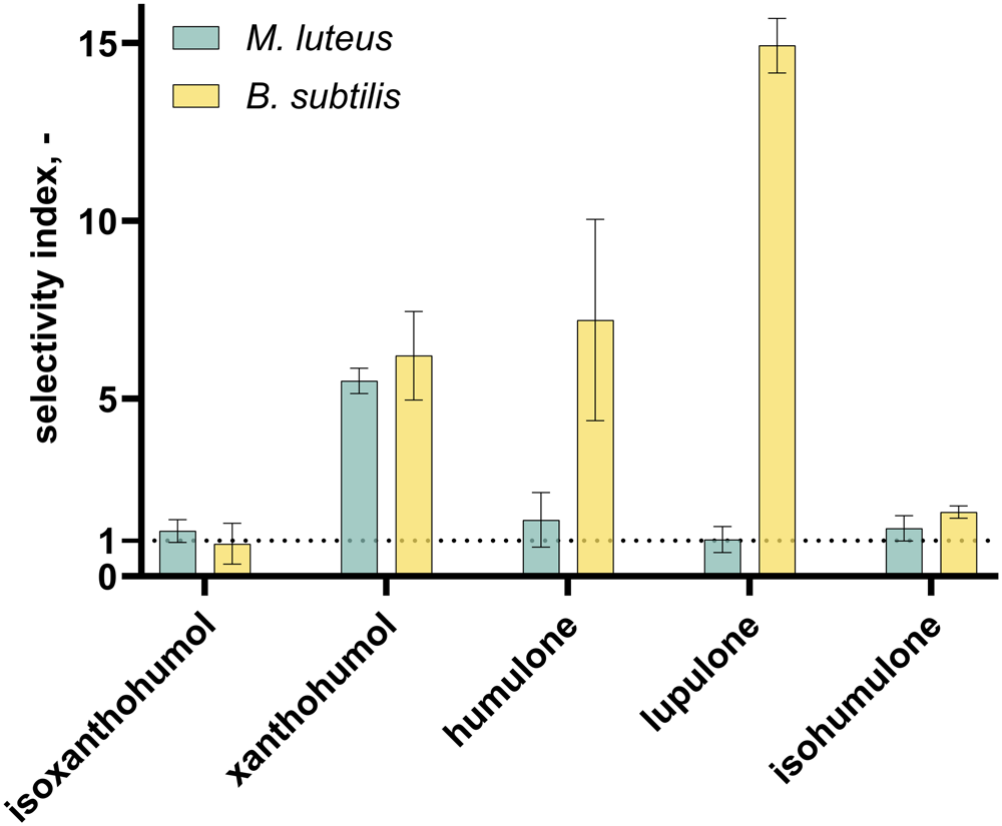
Selective effects of five hop compounds regarding their antibacterial versus cytotoxic properties. Selectivity indices are biological duplicates. *B. subtilis*, *Bacillus subtilis*; *M. luteus*, *Micrococcus luteus*.

Cytotoxic and antibacterial effects need to be considered in relation to each other for a better evaluation of the individual hop compounds. Therefore, the quotient of IC_50_ and MIC_50_ values was calculated as selectivity index (SI), with a value greater than 1 indicating a stronger antibacterial than cytotoxic effect. Results are shown in Figure 9.

It can be concluded that there are also differences in the SI, based on the differing inhibitory effects on the two bacterial strains. In general, all five hop components achieve values around or above the threshold of 1. For *M. luteus*, only xanthohumol was able to achieve a highly selective index (5.5 ± 0.34), which means that it is 5.5 times more antibacterial than cytotoxic. In addition to a similar index around 6 for xanthohumol and humulone for *B. subtilis*, the SI of lupulone is of note, with a value of 15 ± 0.77 that must be highlighted. The isomerized components of hops could not reach favorable indices at all. It is therefore recommended that fresh hop material be utilized in the ensuing analysis, given that spent material from the brewing industry contains isomerized components due to the high temperatures at which it is processed.

## 4 Discussion

### 4.1 Hydrophobic Properties of Hop Pure Compounds

In this study, the solubility of five hop pure compounds was enhanced when using DMEM with 10% FBS h.i. content in comparison with NB and TSB media. This could be attributed to the role of serum as a solubility enhancer (Liu et al. 2023). However, even in the absence of serum, an increase in solubility was still observed compared with the NB and TSB media. This finding suggests that the specific composition of the media, including its salts, sugars, and other carbon sources, strongly influenced the solubility of the individual hop substances. This underscores the necessity for precise monitoring and quantification of concentrations via HPLC for the different media types.

Although all compounds possess hydrophobic properties, humulone, lupulone, and isohumulone showed a solubility limit that is near the concentration of 1 mg mL^−1^ in the media selected, but only a small proportion of xanthohumol could be recovered in the aqueous solvent. Its isoform, isoxanthohumol, is moderately dissolved in the two media because its flavanone structure provides better hydrogen bonding and thus reduced hydrophobicity compared with the chalcone structure of xanthohumol. The limited availability of xanthohumol in aqueous media could be attributed to a combination between hydrophobicity and additional its different polar nature in comparison to humulone and lupulone (PubChem 2025a, 2025b).

### 4.2 Hop Compounds Inhibit Growth of *M. luteus* and *B. subtilis*

To categorize the MIC_50_ values determined, it is necessary to consider that the literature usually refers to MIC_90_ values. MIC_90_ represents 90% inhibition of bacterial growth, whereas MIC_50_ values indicate the concentration at which 50% of bacterial growth is inhibited. In this study, the MIC_50_ value was selected to facilitate a more accurate comparison with the IC_50_ determined in parallel for cytotoxicity testing. In the present study, the antibacterial activity of five hop substances against the bacteria *M. luteus* and *B. subtilis* was investigated. The results demonstrated that all five substances achieved activities in the low to medium µg mL^−1^ range. Of particular note were xanthohumol and lupulone, which exhibited a comparatively strong antibacterial effect.

With a few exceptions, isolates from hops, such as the compounds tested here, are primarily effective against Gram‐positive bacteria (Bocquet et al. 2018), such as *M. luteus* or *B. subtilis*. The efficacy of bitter acids or xanthohumol is dependent on the particular bacterial strain, with the majority of MIC values falling within the same concentration ranges as mentioned above (Bocquet et al. 2018). Gentamicin sulfate showed a comparable value size compared with the tested hop isolates. In addition hop compounds exhibited enhanced antibacterial activity in comparison to several other conventional antibiotics in literature, including ceftriaxone (MIC_90_ = 500 µg mL^−1^), tetracyclines (MIC_90_ = 315 µg mL^−1^), ampicillin (MIC_90_ = 2500 µg mL^−1^), and streptomycin (MIC_90_ = 500 µg mL^−1^), against *M. luteus* (Seniya et al. 2012). These results support the hypothesis that isolates from hops could be a viable alternative to conventional antibiotics.

### 4.3 Hop Pure Compounds Act Cytotoxic on UMNSAH/DF‐1

Studies about the cytotoxic potential of hop (isolated compounds) are limited and frequently performed on (human) tumor cell lines, which can typically exhibit a more sensitive response compared with healthy cell lines (Matt and Hofmann 2016). The comparison of human with avian cell lines, such as UMNSAH/DF‐1 in this study, poses additional challenges (Paradowska et al. 2022). However, Farag and Wessjohann presented IC_50_ values for humulone (13.2–15.5 µg mL^−1^) and lupulone (2.4–8.1 µg mL^−1^) against human prostate (PC3) and colon (HT29) cancer cell lines (Farag and Wessjohann 2013), which are within the same concentration range as in this study. Both compounds are also known for inhibiting cancer cell proliferation for MDA‐MB‐231 (breast cancer) and SK‐MES (lung cancer) cell lines in similar concentrations (Tyrrell et al. 2010). Hop bitter acids target cancer cell lines by inducing apoptosis in rapidly growing tumor cells, disrupting mitochondrial membranes and permeability, by inhibiting angiogenesis and inducing certain cytochrome P450 enzymes (van Cleemput et al. 2009). Prenylated flavonoids, including xanthohumol and its isoform, were tested for their antiproliferative effect against MCF‐7 breast cancer cell line and A‐2780 ovarian cancer cell line, where xanthohumol achieved IC_50_ values of 13.3 and 0.52 µM. Isoxanthohumol exhibited a comparable effect on the MCF‐7 cell line, but was 35 times less effective against the A‐2780 cell line. It is hypothesized that the antiproliferative activity of xanthohumol and isoxanthohumol may be attributable to the inhibition of DNA synthesis and inducing apoptosis (Miranda et al. 1999). The inhibitory effect of xanthohumol on normal murine hepatocytes (AML 12 cell line) was much weaker with an IC_50_ value of 211 µM (Ho et al. 2008). Therefore, our findings can be supported by existing literature showing that lupulone has a higher cytotoxic effect than humulone. Xanthohumol and isoxanthohumol showed different trends depending on the cell line used. Unfortunately, the current literature lacks studies that directly compare all components (isoxanthohumol, xanthohumol, humulone, lupulone, and isohumulone) at once.

### 4.4 Antibacterial Effects in Comparison With Cytotoxic Properties

For using hop isolates as a potential alternative to common antibiotics, it is imperative to consider not only the antibacterial effects of the tested hop compounds, but also to compare these findings with the cytotoxic effects. This enables us to find out the level of selectivity exhibited by the compounds in the study, and whether the antibacterial effect can surpass the cytotoxic effect. In the present context, a value greater than 1 indicates that the antimicrobial effect is several‐fold stronger than the cytotoxic effect. The findings of this study indicated that four out of five compounds exhibited comparable antibacterial and cytotoxic properties for *M. luteus*. However, xanthohumol demonstrated a significantly higher index of 5.5 ± 0.34. Klimek et al. (2021) showed bacterial strain‐dependent selectivity indices of xanthohumol between 0.84 and 136, but also higher IC_50_ values of 26.56 µg mL^−1^ for normal human fibroblasts (BJ cells). In a different study with several *S. aureus* strains and cancer cell lines, an average SI of 0.6 (xanthohumol), 0.08 (humulone), and 2.25 (lupulone) was achieved (Bocquet et al. 2019). Therefore, it has been demonstrated that the outcomes of such experiments are strongly dependent upon the test system used. This was also demonstrated in this study, as the values for *B. subtilis* differed clearly from those of *M. luteus*.

Additionally, Gentamicin sulfate was included as a reference standard. However, direct comparison is limited. Although gentamicin sulfate showed a notably higher SI of ~2000 in this study, it should be kept in mind that the use as a feed additive differs greatly from the use as a classic antibiotic to fight ongoing infections. Moreover, gentamicin is not permitted for prophylactic use in livestock, highlighting differences in practical application. Further studies could achieve more favorable selectivity for hop‐based product indices by utilizing more realistic models, such as ex ovo ones. Due to their more complex structure in comparison with in vitro plate assays, they could be less sensitive to antiproliferating effects of hop (Garle et al. 1994). It is also important to note that hop compounds are to be taken orally, so effects such as bioavailability, stability, and different environments in the organism must be taken into account. Egg models have been shown to provide a more realistic insight into transport, barrier, and metabolic effects (Vargas et al. 2007).

## 5 Conclusions

The present study investigated the solubility, antibacterial, and cytotoxic activities of five hop compounds. The solubility of these compounds was best in DMEM with 10% FBS compared with other media used in these studies, particularly for humulone, lupulone, and isohumulone. Xanthohumol and isoxanthohumol generally showed limited solubility in comparison with the above‐mentioned bitter resins due to the combination of their hydrophobic and polar properties. With regard to the antibacterial activity, it was demonstrated that all compounds exhibited inhibition with xanthohumol (MIC_50_ = 0.95 ± 0.040 µg mL^−1^) against *M. luteus* and lupulone (MIC_50_ = 0.10 ± 0.0029 µg mL^−1^) against *B. subtilis*, exhibiting the strongest effects. With reference to the study's findings on the potential for these compounds to induce toxicity, it was observed that lupulone demonstrated the strongest cytotoxic effect on the UMNSAH/DF‐1 chicken cell line used, with an IC_50_ value of 1.5 ± 0.033 µg mL^−1^. A comparison of the antibacterial and cytotoxic effects revealed that xanthohumol exhibited the highest SI for *M. luteus*, demonstrating 5.5‐fold greater antibacterial activity compared with its cytotoxic effects. For *B. subtilis*, lupulone showed the highest selective effect with an index of around 15. These findings suggest that hop‐derived compounds, particularly xanthohumol, lupulone, and humulone, have the potential to serve as alternatives to conventional antibiotics for the production of a feed additive. The outcomes provide novel insights into the bioactivity of hop compounds and their application potential. Future studies should focus on compound interaction, testing against a broader range of pathogens, in vivo validation, long‐term safety assessments, and optimization of formulations to translate these compounds into practical and effective feed additives.

## Author Contributions

**Luisa Kober:** conceptualization (equal), writing – original draft (lead), experiments and analysis (lead), writing – review and editing (equal). **Kathrin Castiglione:** conceptualization (equal), writing – original draft (supporting), writing – review and editing (equal).

## Ethics Statement

The authors have nothing to report.

## Conflicts of Interest

The authors declare no conflicts of interest.

## Data Availability

All data generated and analyzed during this study are included in this published article. Further data are available from the corresponding author on reasonable request.
